# Assessment of the stream invertebrate β‐diversity along an elevation gradient using a bidimensional null model analysis

**DOI:** 10.1002/ece3.9135

**Published:** 2022-08-04

**Authors:** Pablo Timoner, Pierre Marle, Emmanuel Castella, Anthony Lehmann

**Affiliations:** ^1^ enviroSPACE Group, Department F.‐A. Forel for Environmental and Aquatic Sciences Institute for Environmental Sciences, University of Geneva Geneva Switzerland; ^2^ Aquatic Ecology Group, Department F.‐A. Forel for Environmental and Aquatic Sciences Institute for Environmental Sciences, University of Geneva Geneva Switzerland

**Keywords:** aquatic macroinvertebrates, biodiversity, nestedness, null model, spatial distribution, stochasticity, Swiss Alps, turnover

## Abstract

β‐Diversity, commonly defined as the compositional variation among localities that links local diversity (*α*‐diversity) and regional diversity (*γ*‐diversity), can arise from two different ecological phenomena, namely the spatial species turnover (i.e., species replacement) and the nestedness of assemblages (i.e., species loss). However, any assessment that does not account for stochasticity in community assembly could be biased and misinform conservation management. In this study, we aimed to provide a better understanding of the overall ecological phenomena underlying stream β‐diversity along elevation gradients and to contribute to the rich debate on null model approaches to identify nonrandom patterns in the distribution of taxa. Based on presence‐absence data of 78 stream invertebrate families from 309 sites located in the Swiss Alpine region, we analyzed the effect size of nonrandom spatial distribution of stream invertebrates on the β‐diversity and its two components (i.e., turnover and nestedness). We used a modeling framework that allows exploring the complete range of existing algorithms used in null model analysis and assessing how distribution patterns vary according to an array of possible ecological assumptions. Overall, the turnover of stream invertebrates and the nestedness of assemblages were significantly lower and higher, respectively, than the ones expected by chance. This pattern increased with elevation, and the consistent trend observed along the altitudinal gradient, even in the most conservative analysis, strengthened our findings. Our study suggests that deterministic distribution of stream invertebrates in the Swiss Alpine region is significantly driven by differential dispersal capacity and environmental stress gradients. As long as the ecological assumptions for constructing the null models and their implications are acknowledged, we believe that they still represent useful tools to measure the effect size of nonrandom spatial distribution of taxa on β‐diversity.

## Graphical abstract

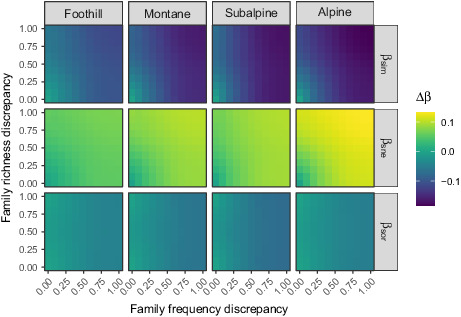

## 1 INTRODUCTION

β‐Diversity, first introduced by Whittaker (1960), is commonly defined as the compositional variation among localities that links local diversity (α‐diversity) and regional diversity (γ‐diversity). In its original form (Whittaker, 1960, 1972), it represents a key concept for biodiversity, as inventory diversity (e.g., number of species), at any spatial scale, depends on the average inventory at the finer‐scale units and on the variation among these units (i.e., β‐diversity). The relationship between the three diversity components (i.e., α, β, and γ) can be multiplicative (β=γ/α) or additive (β=γ+α). However, it was shown that the multiplicative formulation is the only way to obtain independent α and β, when referring to “true diversity” or effective number, which is the number of equally common species required to give a particular value of a diversity index (Baselga, 2010a; Jost, 2007). There exists a welter of methods to calculate β‐diversity, albeit little guidelines for selection (Jost, 2007; Jurasinski et al., 2009; Koleff et al., 2003; Tuomisto, 2010a, 2010b; Vellend, 2001). These methods include metrics directly based on the three diversity components (Jost, 2007; Veech & Crist, 2010), on two‐site (Jaccard, 1912; Simpson, 1943; Sørensen, 1948) or multiple‐site (Baselga et al., 2007; Diserud & Ødegaard, 2007) similarity measures, and on multivariate analyses based on dissimilarity matrices (Anderson et al., 2006; Legendre, 2008). These approaches are conceptually close, as similarity (or dissimilarity) measures are monotonic transformations of the multiplicative Whittaker's β‐diversity (Diserud & Ødegaard, 2007; Jost, 2007).

Two main ecological phenomena produce β‐diversity, namely the spatial species turnover and the nestedness of assemblages, both resulting from species replacement and species loss, respectively (Harrison et al., 1992). A high turnover is mainly related to a strong environmental filtering (Chase et al., 2011; Melo et al., 2009; Wen et al., 2016), while differential dispersal capacity and/or environmental stress gradients usually promote nested assemblages (Baselga, 2010b). Routines partitioning β‐diversity into turnover and nestedness have been proposed (Baselga, 2010b; Schmera et al., 2020) as a basis for associating these components to ecological processes and identifying causal links.

β‐Diversity usually arises from both deterministic (i.e., realized niche‐related) and stochastic processes. However, identifying the nonrandom component is challenging. Null model approaches have been developed for this purpose, enabling comparisons between the observed structural pattern and those obtained randomly, under certain conditions (Gotelli & Graves, 1996). They allow to determine whether the observed β‐diversity deviates from null expectations, and if so, how much. Although these approaches gained popularity (Chase et al., 2011; Dantas de Miranda et al., 2019; Gotelli & Ulrich, 2012; Kraft et al., 2011), the construction of null models remains currently debated, specifically regarding the components of the dataset that should be preserved (e.g., species richness and/or species occurrence), while randomizing the pattern of interest (Connor & Simberloff, 1979; Ulrich & Gotelli, 2013). Randomization of presence‐absence matrices is constrained, at least, by the observed number of rows and columns, which depend on the number of sites and species. Usually, the matrix fill, which corresponds to the ratio of the total number of occurrences to the number of matrix cells, is also fixed. Therefore, the variation among existing null models generally depends on the way marginal totals are treated. For rows corresponding to sites and columns to species, the row and column marginal totals are unconstrained, when assuming that all sites are potentially equally suitable, and all species are potentially equally common, respectively. In contrast, marginal totals are fixed, when assuming species richness and frequencies reflect intrinsic ecological (e.g., habitat harshness) and biological (e.g., reproductive strategy) factors, respectively. Finally, halfway between both options, marginal totals of the null matrix can be proportionally constrained, matching on average those of the original matrix. Procedures have been developed to randomize presence‐absence matrices, using one of these three approaches for rows and columns separately, giving rise to nine different algorithms (Gotelli, 2000). There are unavoidable tradeoffs between Type I (i.e., rejecting a true null hypothesis) and Type II (i.e., failing to reject a false null hypothesis) statistical errors. The least restrictive algorithms are prone to Type I error and the most restrictive prone to Type II error. Because differences in imposed constraints can result in contrasted null model matrices, Strona et al. (2018) proposed a new modeling framework based on a flexible matrix randomization algorithm. The procedure allows exploring the complete null space of existing algorithms and assessing how the magnitude of the structural patterns varies according to a continuous range of possible assumptions.

Multiple studies assessed the variation in β‐diversity along altitudinal gradients (Jankowski et al., 2009; Syfert et al., 2018; Tang et al., 2012). Some accounted for β‐diversity partition (Bishop et al., 2015; Da Silva et al., 2018; Fontana et al., 2020; García‐Navas et al., 2020) and stochasticity (Bishop et al., 2015; Da Silva et al., 2018; Sabatini et al., 2018; Tello et al., 2015). Relatively few assessments focused on stream biodiversity (Castro et al., 2019; Jacobsen, 2003; Jaramillo‐Villa et al., 2010). These all showed a positive relationship between β‐diversity and elevation. However, none of them considered either the two components of β‐diversity separately, or the nonrandom spatial species distribution. Exhibiting high among‐site variation, headwaters have been considered as vital to stream network biodiversity (Finn et al., 2013). However, a better understanding of the ecological phenomena underlying stream β‐diversity is needed for proper inferences. As a matter of fact, higher β‐diversity does not necessarily lead to higher γ‐diversity, especially when it is driven by species loss. Environmental filtering and dispersal limitations occurring at different scales have been suggested to be the main ecological phenomena causing β‐diversity. Similar environmental characteristics are usually related to similar species composition, as long as there are no major accessibility constraints (Freestone & Inouye, 2006). In a null model approach, these deterministic mechanisms should lead to a deviation from the null model, which is only driven by stochasticity. By assessing the relative importance of the turnover and the nestedness of aquatic invertebrates in the Swiss Alpine region taking into account potential stochasticity, we aimed to clarify previous research outcomes and to contribute to the rich debate on the use of null model approach.

## 2 METHODS

### 2.1 Study area and invertebrate data

The Swiss Alpine region occupies an area of 25,819 km^2^ and shows a steep altitudinal gradient (range: 194–4634 m a.s.l.). Climatic conditions are mainly oceanic in the Northern Alps, and Mediterranean in the Southern Alps, while the Central Alps have an intra‐alpine dry continental climate (MeteoSwiss, 2020). The Alpine föhn is a warm wind, which causes warming and drying of air on the lee side of the mountain range and orographic precipitation on the windward slopes. Climate in intra‐alpine valleys is thus relatively mild, whereas higher altitudes experience arctic temperatures.

Presence‐absence data of aquatic invertebrates identified at the family level from 309 sites were obtained from the Swiss Centre for the Cartography of the Fauna (InfoFauna—CSCF). Despite its relatively coarse resolution, the family level has already been used in other β‐diversity assessments (Alves et al., 2020; Da Silva et al., 2018) and it has been shown to be an effective taxonomic surrogate to detect spatial differences in β‐diversity (Terlizzi et al., 2009). As consumers at intermediate trophic levels, aquatic invertebrates are affected by both bottom‐up and top‐down drivers, providing a broad insight into stream ecosystem function (Wallace & Webster, 1996). Furthermore, these organisms show a large spectrum of responses to different stressors, associated with a high taxonomical and functional diversity, and they are relatively sedentary, which allows effective spatial analyses (Rosenberg & Resh, 1993). Observations corresponded to surveys carried out across the Swiss Alpine region between 2013 and 2017 within the framework of the Swiss Biodiversity Monitoring Program. We assumed a relatively high stability of invertebrate assemblages at that temporal scale (Fritz & Dodds, 2004; Lawrence et al., 2010). Standard sampling within streams occurred during the optimal period in terms of invertebrate diversity, from March to June depending on the elevation (Stucki, 2019). We assigned each site to one of four bioclimatic stages (Prunier et al., 2013), which were based on mean annual temperature (MeteoSwiss, 2019) and distributed along the altitudinal gradient (Table 1).

**TABLE 1:** Four bioclimatic stages used in this study, based on the mean annual temperature averaged over the years 2009–2018 (MeteoSwiss, 2019). The elevation range corresponds to the sampling elevation range of the observed data for each bioclimatic stage

Bioclimatic stage	Mean temperature	Elevation range
Foothill	>8°C	196–1203 m a.s.l.
Montane	4–8°C	582–2174 m a.s.l.
Subalpine	0–4°C	1314–2555 m a.s.l.
Alpine	<0°C	2036–2631 m a.s.l.

**FIGURE 1:**
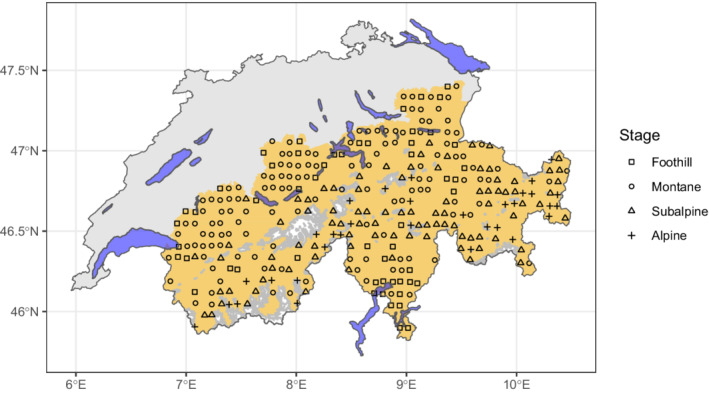
Map of Switzerland and the location of the observations in the Swiss Alpine region (*N* = 309). Symbols relate to the bioclimatic stage of each observation. The golden and purple shades represent the Swiss Alpine region and lakes, respectively.

**FIGURE 2:**
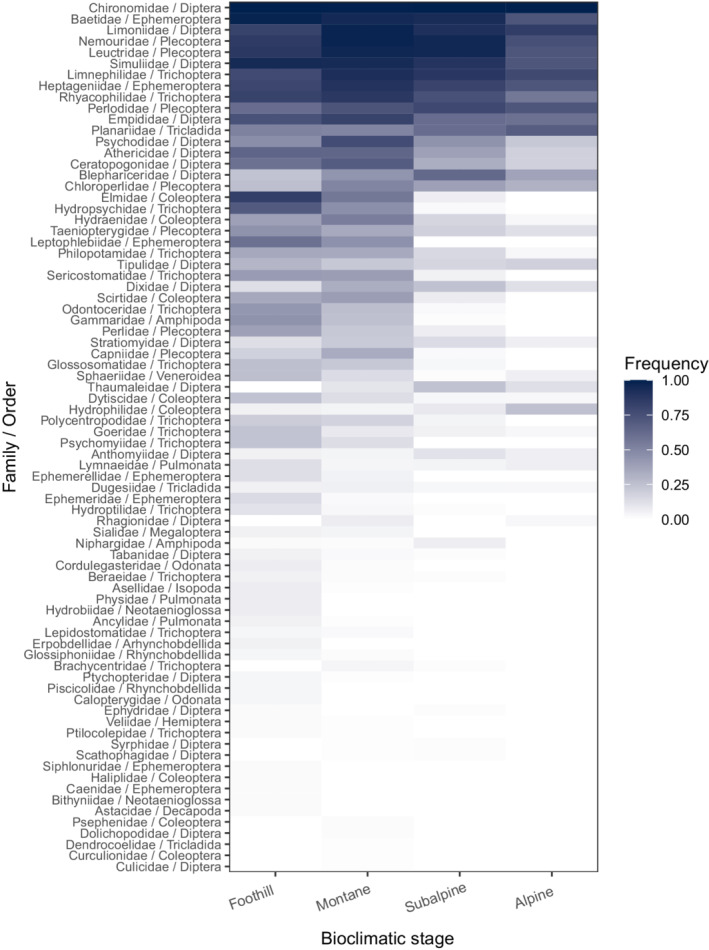
Heatmap showing the observed taxon frequency at the four bioclimatic stages in the Swiss Alpine regions.

As the study area included different slope orientations, we preferred to use temperature rather than elevational classes to reduce the bias caused by potential differences in environmental condition heterogeneity. Median pairwise Euclidean distances between sites among stages ranged from 80 to 99 km. Distances were slightly lower at the subalpine stage, but no gradual changes were observed. The number of sampled sites varied among the stages (Figure 1); however, no major sampling effort bias was suspected, as this number followed the distribution of stream length across elevation, peaking at medium altitude. Nevertheless, to facilitate the comparison through the bioclimatic stages and reduce a potential bias caused by these differences, we randomly selected the same number of sites per level, corresponding to the minimum number of observations among the stages (i.e., 32 sites). This procedure, as well as the subsequent analyses, was repeated 100 times, and the results were averaged to produce a single estimation per level. The 78 taxonomical families belonging to 17 taxonomical orders taken into account in this study are listed with their observed frequencies at each bioclimatic stage in Figure 2.

### 2.2 Analysis

#### 2.2.1 β‐Diversity

We used the β‐diversity partition proposed by Baselga (2010b) to assess the overall β‐diversity, as well as its turnover and nestedness components. Considering the Sørensen ($\beta_{\mathrm{sor}}$) and the Simpson ($\beta_{\mathrm{sim}}$) dissimilarity indices as measures of the total β‐diversity and the spatial turnover, respectively, Baselga (2010b) derived the nestedness component ($\beta_{\mathrm{sne}}$), so that $\beta_{\mathrm{sor}}=\beta_{\mathrm{sim}}+\beta_{\mathrm{sne}}$. As pairwise dissimilarities do not account for patterns of co‐occurrence among more than two sites, and therefore, do not accurately reflect the overall compositional heterogeneity (Baselga, 2013; Diserud & Ødegaard, 2007), we opted for the multiple‐site indices. For each bioclimatic stage, we calculated the three indices, as well as two of the most relevant presence‐absence matrix parameters, namely the γ‐diversity, which determines the dimension of the matrix for a fixed number of sites, and the matrix fill. As major ecological assumptions are made when fixing these parameters in null model analysis, we found insightful to assess their relationship with the β‐diversity indices in the context of our data. The β‐diversity indices were calculated using the “betapart” package (Baselga et al., 2018) in R (R Core Team, 2019).

#### 2.2.2 Null model

The effect size of nonrandom spatial distribution of taxa on β‐diversity, also called β‐deviation (Δβ), was assessed using the “Tuning Peg” (TP) algorithm from Strona et al. (2018). In combination with a wise random walk, it allows exploring the complete null space, which is subdivided in a regular two‐dimensional grid containing 121 cells (11 × 11) with the two dimensions corresponding, respectively, to the discrepancy in row and column marginal totals between the original and the randomized matrix. At the bottom‐left corner of the null space, we have the most conservative algorithm that preserves the row and column marginal totals, and at the other extreme, the most liberal one, which places occurrences in cells with equal probability. We applied the TP algorithm to our original matrices, and we calculated the deviation from the null expectation at each node of the grid. As $\beta_{\mathrm{sor}}$, $\beta_{\mathrm{sim}}$, and $\beta_{\mathrm{sne}}$ are in the same unit, ranging all between 0 and 1, and in order to preserve their additive property, we opted to consider the raw instead of the standardized β‐deviation. For each index, the deviation was calculated as the difference between the observed β ($\beta_{\mathrm{obs}}$) and the mean of the null distributions ($\bar{\beta_{\text{null}}}$):

(1) $$\Delta \beta =\beta_{\mathrm{obs}}-\bar{\beta_{\text{null}}}.$$

Positive and negative values indicate a higher and a lower β‐diversity, respectively, than the one expected by chance. We repeated the procedure 100 times, and for each node, we averaged the deviations and calculated *p*‐values computed as the fraction of null matrices having β‐diversity scores higher than the original ones. We created two landscapes of β‐deviation and significance, by plotting the deviation and the *p*‐values for each index and bioclimatic stage in a bidimensional grid corresponding to the null space. Considering a level of significance of .05, β‐deviation was significant when its corresponding *p*‐value was lower than .05 or higher than .95. We compared the $\beta_{\mathrm{sor}}$‐, $\beta_{\mathrm{sim}}$‐, and $\beta_{\mathrm{sne}}$‐deviations among the bioclimatic stages, taking into account different levels of conservativeness. The β‐deviation obtained within the bottom‐left quarter (i.e., relatively low discrepancy) and the top‐right quarter (i.e., relatively high discrepancy) of the bidimensional null space was considered as conservative and liberal, respectively, while considering the overall results as moderately conservative. Finally, pairwise Wilcoxon tests were used to assess the differences among the bioclimatic stages (α level = 0.05), for each level of conservativeness, using the Holm procedure to control the family‐wise error rate. For the TP algorithm, we used a slightly modified version of the R script from Strona et al. (2018) (i.e., we replaced the diversity indices), in order to take into account the β‐diversity indices from Baselga (2010b).

## 3 RESULTS

**FIGURE 3:**
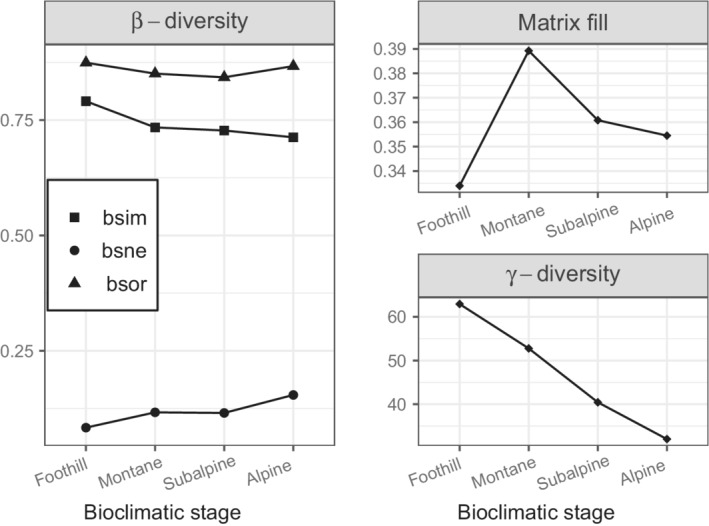
Total β‐diversity ($\beta_{\mathrm{sor}}$) and its components ($\beta_{\mathrm{sim}}$ and $\beta_{\mathrm{sne}}$), as well as the γ‐diversity and the matrix fill along the altitudinal gradient, based on observed invertebrate data. The points represent the average values at each bioclimatic stage.

Overall, the family spatial turnover contributed much more than nestedness to the total β‐diversity. The very high total β‐diversity was relatively stable along the altitudinal gradient, whereas the spatial turnover and the nestedness components decreased and increased, respectively, with elevation (Figure 3). These trends were not related to the variation in the matrix fill, which fluctuated along the altitudinal gradient, within a narrow range of values. In contrast, we observed a regular trend of the γ‐diversity, which decreased regularly from low to high elevation.

**FIGURE 4:**
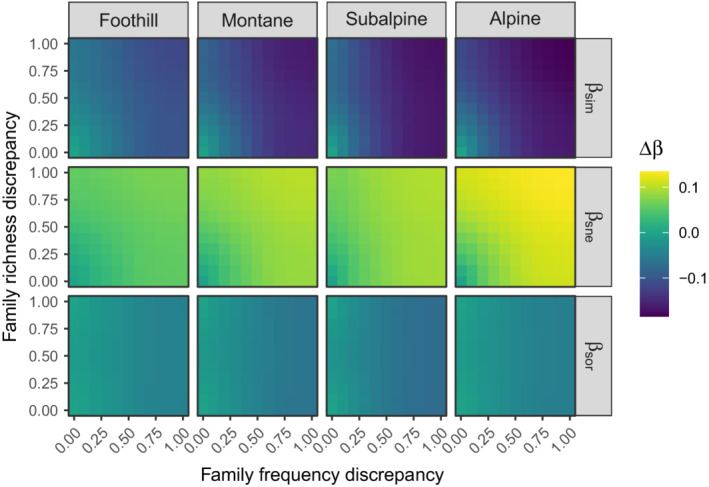
Effect size of nonrandom spatial distribution of taxa on the total β‐diversity ($\beta_{\mathrm{sor}}$), and on its spatial turnover ($\beta_{\mathrm{sim}}$) and nestedness ($\beta_{\mathrm{sne}}$) components, measured as the deviation from the null expectation, for different values of discrepancy in row and column marginal totals between the original and the randomized matrix.

**FIGURE 5:**
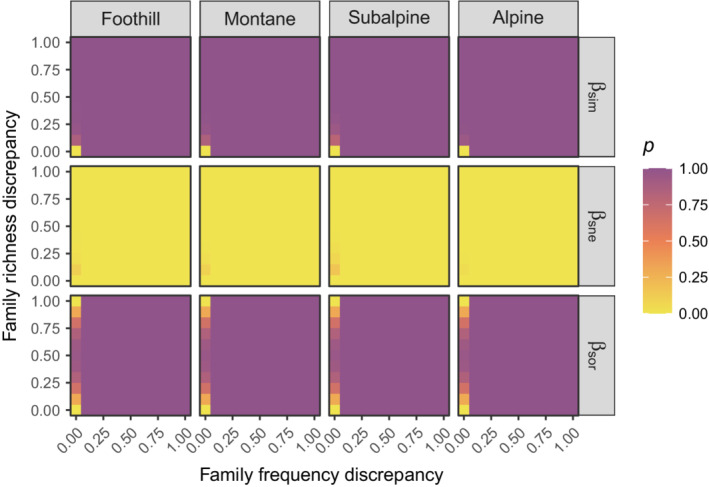
Significance of the effect size of nonrandom spatial distribution of taxa on the total β‐diversity ($\beta_{\mathrm{sor}}$), and on its spatial turnover ($\beta_{\mathrm{sim}}$) and nestedness ($\beta_{\mathrm{sne}}$) components, for different values of discrepancy in row and column marginal totals between the original and the randomized matrix. *P*‐values were computed as the fraction of null matrices having β‐diversity values higher than the original ones. Considering a level of significance of .05, the effect size was significant when its corresponding *p*‐value was lower than .05 or higher than .95.

The effect size of nonrandom spatial distribution of taxa on the total β‐diversity was always negative but close to zero. The slight variations, for a given bioclimatic stage, mainly depended on how the family frequencies were treated in the null model (Figure 4). The higher the discrepancy, the more negative the deviation. Except when the family frequencies were fixed, deviation from the null model was always significant (Figure 5). The effect size of nonrandom distribution was much more pronounced for $\beta_{\mathrm{sim}}$ and $\beta_{\mathrm{sne}}$, which behaved in opposite directions (Figure 4). While $\beta_{\mathrm{sim}}$‐deviation was always negative, $\beta_{\mathrm{sne}}$‐deviation was always positive, and both discrepancies in family frequencies and richness had an influence on the absolute deviations. Except for very constrained null models, the measured effect sizes were always significant (Figure 5).

**FIGURE 6:**
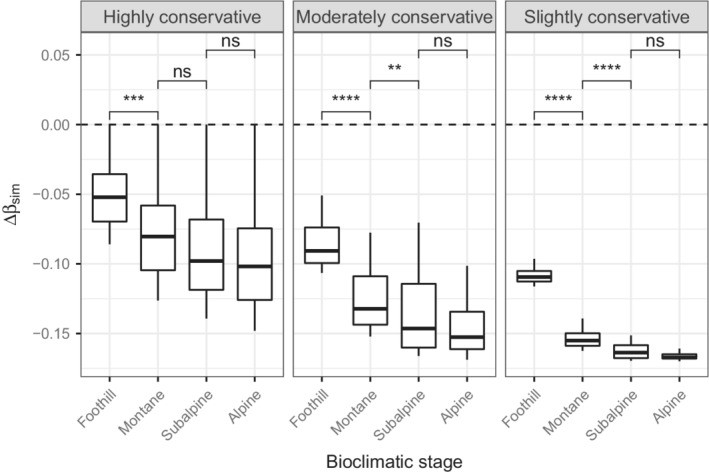
Distribution of the $\beta_{\mathrm{sim}}$‐deviation ($\Delta \beta_{\mathrm{sim}}$) along the altitudinal gradient, according to different levels of conservativeness. Symbols located above the brackets indicate the difference significance between consecutive bioclimatic stages (**p ≤ .01, ****p ≤ .0001, ns: nonsignificant). The horizontal dashed line represents the null expectation. Positive and negative values indicate a stronger and a weaker pattern, respectively, than the one expected by chance.

**FIGURE 7:**
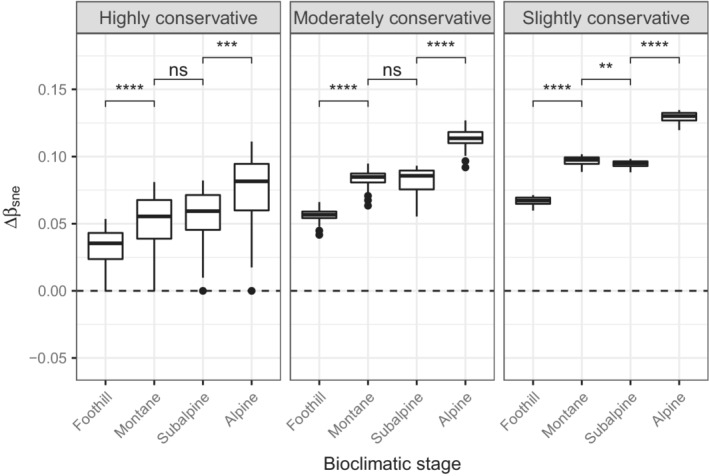
Distribution of the $\beta_{\mathrm{sne}}$‐deviation ($\Delta \beta_{\mathrm{sne}}$) along the altitudinal gradient, according to different levels of conservativeness. See Figure 6 for more details regarding the symbols and the horizontal dashed line.

**FIGURE 8:**
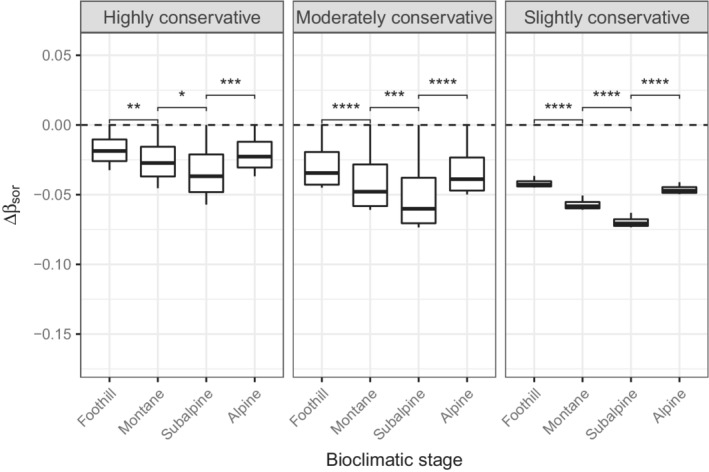
Distribution of the $\beta_{\mathrm{sor}}$‐deviation ($\Delta \beta_{\mathrm{sor}}$) along the altitudinal gradient, according to different levels of conservativeness. See Figure 6 for more details regarding the symbols and the horizontal dashed line.

Overall, the absolute deviations of $\beta_{\mathrm{sim}}$ and $\beta_{\mathrm{sne}}$ from the null model tend to increase with elevation (Figures 6 and 7). The $\beta_{\mathrm{sim}}$‐deviation declined along the altitudinal gradient, while the opposite trend was observed for $\beta_{\mathrm{sne}}$. Differences in deviation between the subalpine and the alpine stages for $\beta_{\mathrm{sim}}$ were not significant, regardless of the level of conservativeness. In contrast, differences for $\beta_{\mathrm{sne}}$ between these stages were always significant. Differences in deviation among the bioclimatic stages for $\beta_{\mathrm{sor}}$ were always significant, with absolute values being higher at the subalpine stage (Figure 8). Finally, the more constrained, the higher the deviation variance.

## 4 DISCUSSION

### 4.1 Raw β‐diversity

In contrast with other studies that focused on aquatic invertebrates (Castro et al., 2019; Jacobsen, 2003), we observed no increase in the total β‐diversity along the altitudinal gradient. Assuming a negative relationship between stream order and elevation, our results also differed from those of Finn et al. (2013), who showed that headwaters were associated with higher β‐diversity than mid‐order streams. They also used the Sørensen's dissimilarity index, which supposedly makes our studies comparable. However, their outcomes were based on mean pairwise dissimilarities instead of the multiple‐site index. Both methods can yield different results and lead to contrasted ecological inferences. Indeed, using pairwise dissimilarities in a supplementary analysis, we observed, in line with Finn et al. (2013), that the raw $\beta_{\mathrm{sor}}$ was much higher in the alpine than in the subalpine or montane stages. Nevertheless, unlike the multiple‐site index, pairwise dissimilarities do not account for patterns of co‐occurrence involving more than two sites, and they are not linked to the strict sense definition of β‐diversity, namely the effective number of different communities in the regional pool (Baselga, 2013). Regarding the contribution of the two β‐diversity components, our results matched those obtained in assessments that focused on macrophytes (Alahuhta et al., 2017; Fu et al., 2019), highlighting the prominent role of the spatial turnover in explaining the total β‐diversity. Nonetheless, any ecological interpretation of this pattern that ignores the potential random processes in community assembly could be biased and misinform conservation management. Indeed, while preserving taxon‐rich areas may be recommended in systems structured by biotic nestedness, a network of small reserves is advisable to protect most of the taxa where the spatial turnover is high (Clarke et al., 2008).

### 4.2 β‐deviation: ecological and methodological considerations

We observed a particularly low $\beta_{\mathrm{sor}}$‐deviation, yet overall significant, which means that the observed $\beta_{\mathrm{sor}}$ was close to the null expectation. This was not surprising, as $\beta_{\mathrm{sor}}$, being a monotonic transformation of the multiplicative Whittaker's β‐diversity, mainly depends on the matrix fill (Ulrich et al., 2017), which was fixed for the construction of the null models. This highlights the importance of being aware of the null model constraints and its implications. In a null model analysis, the measured effect size of nonrandom factors only relates to ecological processes that actually influence the varying parameters, but not the fixed ones. Therefore, the $\beta_{\mathrm{sor}}$‐deviation only reflected a minor part of the ecological processes producing the total β‐diversity, that is, the one that is not related to the matrix fill. Although our null model analysis may be irrelevant for $\beta_{\mathrm{sor}}$, it provided important insights into the effect size of nonrandom spatial distribution of taxa on its components, namely the turnover and the nestedness. These are mostly related to the distribution of the marginal totals and do not depend on the matrix fill, at least in our case. Indeed, while nestedness structures cannot occur with rare species only (i.e., very low matrix fill), both turnover of taxa and nested assemblages are possible considering the mean frequency of the taxa included in this study (≈0.33). That being said, the spatial turnover of invertebrate families and the nestedness of assemblages were notably lower and higher, respectively, than the ones expected by chance. We believe that both the large topographic barriers existing in the Alps (Tonkin et al., 2018) and the fragmentation of habitats induced by human activities are limiting the dispersal of some aquatic invertebrates in the Swiss Alpine region (Dynesius & Nilsson, 1994; Ward & Stanford, 1995). Dam constructions represent a neat example of how stream habitats can be fragmented, but depending on how the aquatic invertebrates disperse (Bohonak & Jenkins, 2003; May, 2019), human activities in the catchment can also undermine inland connectivity. Furthermore, at high altitude, the stream conditions become harsh (Birrell et al., 2020; Jacobsen & Dangles, 2017), and the network distances between streams are larger due to the dendritic structure of the river network. At lower elevation, rivers and streams suffer pollution from urban and agricultural areas (Kunz et al., 2016). Stressful conditions and differential dispersal capacity are known to promote higher nestedness structures (Baselga, 2010b) and to reduce the relative contribution of the taxon turnover to the total β‐diversity.

Based on the raw index scores, we could infer that the sole variation in γ‐diversity may explain the differences in $\beta_{\mathrm{sim}}$‐ and $\beta_{\mathrm{sne}}$‐deviation along the altitudinal gradient. However, after accounting for it in the null model analysis, we still observed important changes in both components of the β‐diversity, regardless of the level of conservativeness. At high elevation, the lower and the higher $\beta_{\mathrm{sim}}$‐ and $\beta_{\mathrm{sne}}$‐deviation, respectively, could be explained by the effects of glacier melt contribution to stream runoff, acting as a stronger filter. We also hypothesize that, following the general theory of the abundance–occupancy relationships (Gaston et al., 2000), potential dispersal limitations promoting nestedness might be linked to the low abundance of the majority of the taxa at high elevation in the Swiss Alpine region (Alther et al., 2019). In contrast, at low elevation, the relatively higher and lower $\beta_{\mathrm{sim}}$‐ and $\beta_{\mathrm{sne}}$‐deviation, respectively, might be related to a potential higher “natural” environmental heterogeneity and the broader range of human impact types (Kunz et al., 2016). The diversity of anthropogenic influences may produce a diversity of ecological responses that are unlikely to be observed at high altitude, potentially strengthening the environmental filtering. A single disturbance promotes nestedness of assemblages, but different kinds of disturbance (e.g., eutrophication, pesticide and industrial pollution, and habitat degradation) in different sites could, potentially, enhance the taxon turnover. In human‐impacted regions, the variation in β‐diversity along the altitudinal gradient is, thus, very likely to be driven by a complex interaction between “natural” and anthropogenic factors.

Overall, the raw index scores and the deviation values followed analogous trends across elevation, which means that the null model parameters at each biological stage were sufficiently close to produce similar random patterns, leading to relatively constant null values. The conditions were clearly favorable toward high turnover, which made the actual nestedness pattern remarkable. Furthermore, the lower the constraints were, the lower the null model variance was, and the closer the trends of both the raw and the deviation values were. This underlines the high consistency of the three indices under flexible randomization.

### 4.3 Study limitations

We are aware of the limitations of our study, particularly those regarding site distribution. Indeed, these were not distributed along a single mountainside, but across the entire Swiss Alpine region. Even though we opted to consider bioclimatic stages instead of elevation, our results were potentially affected by influence of the spatial heterogeneity at this scale. At least, the distances among sites were quite similar regardless of elevation, and we observed no indication of a possible relationship between our results and the small extent differences among the analyzed bioclimatic stages. Furthermore, the taxonomic resolution was relatively coarse, and the considered taxa had a potentially large range of suitable habitats. This might partially explain the relatively low spatial turnover. Even if families have been used in other studies on β‐diversity (Alves et al., 2020; Da Silva et al., 2018) and shown to be effective taxonomic surrogates to detect spatial differences in β‐diversity (Terlizzi et al., 2009), processes occurring at the species level may not be reflected in our analysis.

### 4.4 Null model debate

Beyond the question of the boundary conditions, Ulrich et al. (2017) argued that null model analysis approaches are unable to determine whether measured β‐deviation evidences real ecological processes or just differences in the size of the species pool. In our opinion, the size of the species pool per se does not represent any problem when assessing β‐diversity. Multiplicative β‐diversity and all its monotonic transformations are not intrinsically dependent on γ‐diversity (Baselga, 2010a). The actual issue lies in the bias that different scales and/or sampling efforts can induce when analyzing the γ‐ α‐diversity relationship, and we agree that null models do not correct for it. This is not their objective either, and according to Xing and He (2020), this misinterpretation has discredited the use of a promising approach in community ecology. Differences in γ‐diversity from different areas of the same extent and equally sampled should not be a concern. Ulrich et al. (2017) also showed that deviation in the proportional species turnover ($\beta_{p}$) was a simple function of the observed matrix fill. Given the fact that $\beta_{p}$ only depends on the matrix fill and that the randomization parameter allowed the matrix fill to vary, their finding is not surprising. Ultimately, they highlighted that the deviation from the null expectation of a given index is a function of the parameter, in a broad sense, that is allowed to vary when constructing the null model, as far as it influences its score. This may sound logical, but it emphasizes the non‐triviality of the ecological hypotheses regarding the deterministic factors that drive the β‐diversity, as any β‐deviation is calculated based on these hypotheses. In consequence, both the conditions under which the original matrix is randomized and the sensitivity of the chosen metric to these conditions must be carefully selected to be ecologically plausible, and to avoid Type I and II errors (Gotelli, 2000).

In this study, we assumed a fixed γ‐diversity, hypothesizing that it is the spatial distribution of taxa that is responsible for the β‐diversity, and not the γ‐diversity per se. We also assumed a fixed matrix fill, which limited our assessment of the effect size of nonrandom processes on $\beta_{\mathrm{sor}}$, but allowed us to focus on the relative contribution of $\beta_{\mathrm{sim}}$ and $\beta_{\mathrm{sne}}$. Finally, we did not take into account any rationale regarding the family frequencies and the site richness, but rather a continuous range of possible assumptions.

## 5 CONCLUSION

We found that the spatial turnover of invertebrate families and the nestedness of assemblages in the Swiss Alpine region were notably lower and higher, respectively, than the ones expected by chance. This pattern increased with elevation pointing out the fact that the deterministic ecological phenomena underlying stream β‐diversity vary along the elevation gradient. Nested pattern is likely to be promoted by pollution at low altitude and by harsh conditions and accessibility constraints at high altitude. From a methodological perspective and in order to clarify the goals and the limitations of the null model approach and prevent ecological misinterpretation, we encourage other researchers to explicitly state and discuss their own assumptions and their implications, when using null model approaches in ecology.

## AUTHOR CONTRIBUTIONS

**Pablo Timoner:** Conceptualization (lead); formal analysis (lead); methodology (lead); visualization (lead); writing – original draft (lead); writing – review and editing (lead). **Pierre Marle:** Methodology (supporting); visualization (supporting); writing – review and editing (supporting). **Emmanuel Castella:** Methodology (supporting); supervision (equal); validation (equal); writing – review and editing (supporting). **Anthony Lehmann:** Funding acquisition (lead); project administration (lead); supervision (equal); validation (equal); writing – review and editing (supporting).

## CONFLICT OF INTEREST

The authors declare no conflict of interest.

## Data Availability

All the raw data that support the findings of this study are provided at: https://doi.org/10.5061/dryad.6hdr7sr2h. All the code that supports the findings of this study is provided at: https://doi.org/10.5281/zenodo.5799486. R (v.4.1.1) was used for data analysis, including the following packages: betapart (v.1.5.4), plyr (v.1.8.6), igraph (v.1.2.9), reshape2 (v.1.4.4), foreach (v.1.5.1), doParallel (v.1.0.16), ggplot2 (v.3.3.5), ggpubr (v.0.4.0), cowplot (v.1.1.1), and viridis (v.0.6.2). For the “Tuning Peg” algorithm, we used a slightly modified version of the R script from Strona et al. (2018), in order to take into account the β‐diversity indices from Baselga (2010a).
